# Critical Evaluation of Acetylthiocholine Iodide and Acetylthiocholine Chloride as Substrates for Amperometric Biosensors Based on Acetylcholinesterase

**DOI:** 10.3390/s130201603

**Published:** 2013-01-25

**Authors:** Madalina-Petruta Bucur, Bogdan Bucur, Gabriel-Lucian Radu

**Affiliations:** National Institute of Research and Development for Biological Sciences, Bioanalysis Center, 296 Splaiul Independentei, Bucharest 060031, Romania; E-Mails: madalina_dondoi@yahoo.com (M.-P.B.); rglucian2000@yahoo.com (G.-L.R.)

**Keywords:** acetylthiocholine iodide, acetylthiocholine chloride, amperometry, acetylcholinesterase

## Abstract

Numerous amperometric biosensors have been developed for the fast analysis of neurotoxic insecticides based on inhibition of cholinesterase (AChE). The analytical signal is quantified by the oxidation of the thiocholine that is produced enzymatically by the hydrolysis of the acetylthiocholine pseudosubstrate. The pseudosubstrate is a cation and it is associated with chloride or iodide as corresponding anion to form a salt. The iodide salt is cheaper, but it is electrochemically active and consequently more difficult to use in electrochemical analytical devices. We investigate the possibility of using acetylthiocholine iodide as pseudosubstrate for amperometric detection. Our investigation demonstrates that operational conditions for any amperometric biosensor that use acetylthiocholine iodide must be thoroughly optimized to avoid false analytical signals or a reduced sensitivity. The working overpotential determined for different screen-printed electrodes was: carbon-nanotubes (360 mV), platinum (560 mV), gold (370 mV, based on a catalytic effect of iodide) or cobalt phthalocyanine (110 mV, but with a significant reduced sensitivity in the presence of iodide anions).

## 1. Introduction

Various types of biosensors based on inhibition of acetylcholinesterase (AChE) have been developed for the rapid detection of neurotoxic insecticides (organophosphates or carbamates), nerve agents and natural toxins (aflatoxin, glycoalkaloids, *etc.*) [1]. Besides biosensors, the AChE activity was intensively investigated *in vivo* due to its primary role in the nervous system of terminating the nerve impulses by acetylcholine catalysis, but also for its relevance in other important processes such as memory [2] or neurodegenerative diseases [3]. Depending on their measurement principle electrochemical biosensors may be potentiometric [4], conductometric [5] or amperometric. The most widely employed detection is amperometric [1] based on two strategies: (i) the use of a natural substrate (acetylcholine) with a second enzyme (choline oxidase) and measurement of the produced hydrogen peroxide or consumed oxygen [6] and (ii) the use of a pseudosubstrate (acetylthiocholine) and the oxidation of the produced [7]. The biosensors are intended to be simple and cheap analytical devices and, in consequence, the monoenzymatic alternative based on acetylthiocholine is preferred over the bienzymatic detection scheme based on choline oxidase. Acetylthiocholine contains a quaternary ammonium cation and it is associated with chloride or iodide as corresponding anion to form a salt. The iodide salt is cheaper, but iodide is electrochemically active and in consequence it is usually employed in spectrometric detection of AChE activity [8]. Besides AChE, biosensors for insecticide detection may be developed based on an alternative enzyme: butyrylcholinesterase (BuChE) that may use either its natural substrate (butyrylcholine) or the artificial substrate (butyrylthiocholine). The product of the enzymatic hydrolysis of both enzymes is the same (thio)choline and the substrate contains either chloride or iodide as counteranion. Thus, the electrochemical study of thiocholine detection in the presence of iodide is relevant for both types of amperometric biosensors based on AChE or BuChE.

In a comparative study, it was observed that both chloride and iodide substrates may be equally used for potentiometric measurement, but only the chloride substrate was employed for amperometric detection [9]. Nevertheless, there are papers reporting the use of substrate with iodide as counteranion for amperometric detection of cholinesterase activity. This paper investigates the possibility of using the acetylthiocholine iodide as enzymatic substrate for the amperometric detection of cholinesterase activity. One important issue in using iodide containing substrate is the high excess of substrate in comparison with the thiocholine that is produced by enzymatic hydrolysis: the substrate concentration is at mM range while the detected thiocholine is produced in low μM concentrations. Thus, even if the peak oxidation of iodide is at higher overpotential than thiocholine, a parasite analytical signal may be still be generated by iodide impeding the use of this substrate. This analytical signal is due to the oxidation of the iodide, is independent of AChE activity and, in consequence, alters the calculated inhibition percentage and led to the impossibility to observe a total enzyme inhibition.

## 2. Experimental Section

Thiocholine stock solution were daily prepared by the enzymatic hydrolysis of 10 mM acetylthiocholine chloride by 5 IU acetylcholinesterase during 30 min. Potassium iodide stock solutions were prepared daily and protected from light. The phosphate buffer solution (PBS), 0.1 M with pH 7 supplemented with 0.1 M KCl was prepared with Milli-Q ultrapure water (Millipore, Billerica, MA, USA). All the reagents were of analytical grade and purchased from Sigma-Aldrich (St. Louis, MO, USA).

The screen-printed electrodes were produced on a ceramic substrate by DropSens (Oviedo, Spain) and have a circular working electrode (WE) with 4 mm diameter, a crescent shaped auxiliary electrode (AE) around the WE and a Ag/AgCl pseudoreference electrode (RE). We have investigated different WE materials: gold, platinum and carbon inks that are simple or modified with carbon-nanotubes or cobalt phthalocyanine. Biosensors were prepared by carefully spreading on the WE surface 3 μL of a freshly prepared solution containing 0.1% BSA (Albumin from bovine serum fraction V), 0.25% glutaraldehyde and 3 IU/mL AChE from electric eel in PBS.

The cyclic voltammetry (CV) and amperometric measurements were performed with a galvanostat/potentiostat Autolab PGSTAT302N (Metrohm-Autolab, Utrecht, The Netherlands) controlled by a PC with the software Nova 1.8. In amperometry, the electromagnetic noise produced by magnetic stirring was reduced using the filter from the ECD module set to 1 s. The CV were made between −0.4 and 0.8 V at a scan rate of 100 mV/s in PBS. Amperometric measurements were performed at different potentials (depending on the WE material) in magnetically stirred solutions with successive injections of thiocholine or potassium iodide.

## 3. Results and Discussion

### 3.1. Choice of the Measurement Conditions

This study was made using two different electrochemical techniques: CV in order to detect the optimum working potentials in function of WE material and amperometry to mimic the measurements of the AChE activity. All the measurements were made in PBS pH = 7.0, a reaction medium usually used with AChE based amperometric biosensors. One important aspect is the concentrations of thiocholine and potassium iodide (both absolute concentrations and relative one to another) to correctly investigate the electrochemical phenomena that takes place during measurements with AChE biosensors. The CV studies were made with 1 mM solutions of potassium iodide or thiocholine in order to measure the redox peaks, but the amperometric measurements were made by injecting into the electrochemical cell concentrations that are similar with the ones expected during amperometric measurements with AChE biosensors. Thus, it was injected 0.1, 0.5 and 1 mM KI (final concentrations) to measure the signals due to the iodide anions because the acetylthiocholine (chloride or iodide) is used at these concentrations to quantify the AChE activity. The choice of the thiocholine concentration is more difficult. For a simpler system, we assume that the measurements are made with the enzyme free in the solution rather than immobilized on the surface of the WE. In this case, it is used approximately 5–30 mIU AChE in the electrochemical cell. The thiocholine concentration in the cell may be calculated thus: 5 mIU AChE will catalyze the production of 5 × 10^−3^ (corresponding to the enzymatic activity) ×10^−6^ (the activity of one IU of enzyme) = 5 × 10^−9^ moles of thiocholine per min. If the electrochemical cell has a total volume of 1 mL then the thiocholine concentration is 5 × 10^−6^ M. This calculus just allows one to estimate the real concentration and must be adjusted depending on the volume cell and measurement time. For the biosensors based on immobilized enzyme (majority of published applications), the enzymatic product is locally produced near the surface of the WE. The thiocholine concentration must be evaluated on the electrode surface and not in the solution (where it is very low). This is very difficult due to several factors: it is not known the exact activity of the immobilized enzyme (it is known the activity of the enzyme used during the immobilization process, but a large percentage may be lost or denatured), the catalytic activity of the immobilized enzymes is different from one the free in solution (it is influenced by several factors like: the orientation of the enzyme that may induce a sterical hindrance, the supplementary diffusion layers if the enzyme was immobilized by entrapment in a microporous matrix, enzyme structure partial perturbation due to the reticulation), the produced thiocholine is diffusing in two directions (towards the electrode surface, but also directly into the solution evading the detection). In fact, despite the fact that numerous authors used Michaelis-Menten kinetics for immobilized enzymes and calculate apparent affinity constants and maximum reaction rates (K_M_^app^ and respectively V_max_^app^), the immobilized enzyme does not follow Michaelis-Menten kinetics and typical shape of the biosensors response to substrate addition (linear increase followed by a plateau) depends on many factors including substrate diffusion, bulk solution stirring, rate of electron transfer, electrode surface fouling, *etc.*[10]. One possibility avoid the need of complicated mathematical estimation of the thiocholine concentration at the surface of the WE is to use a biosensor and inject thiocholine into solution and choose the concentration that provide an analytical signal similar with the one obtained using the enzymatic substrate (acetylthiocholine). The thiocholine final concentrations used in this study are: 5, 15, 25, 45 and 95 μM. The amperometric measurements were made in two variations: by injecting first the potassium iodide followed by thiocholine (to mimic the real measurements with the biosensor were into the electrochemical cell is present first the substrate in large concentration and subsequently appears the thiocholine in increasing concentrations) and by injecting the thiocholine before potassium iodide (to record the analytical signal unperturbed by the iodide).

### 3.2. Carbon and MWCNT Screen-Printed Electrodes

**Figure 1.**
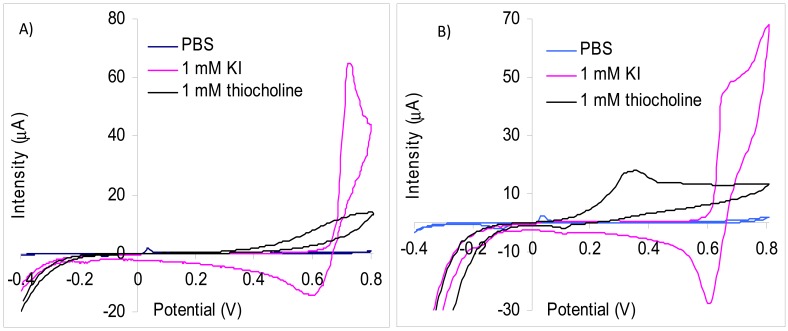
The cyclic voltammograms obtained in PBS, iodide and thiocholine solutions using: (**A**) carbon screen-printed electrode. (**B**) CNT screen-printed electrode.

We have used screen-printed electrodes in our experiments because the enzymatic biosensors based on inhibition are single use. There are numerous types of inks for screen-printing and their exact composition is usually not given by the producers. The screen-printed electrodes are further modified with membranes or by different surface activation/cleaning procedures and thus the experimental results are not always directly comparable with the data published in literature. Our results of the CV study carried out with carbon screen-printed electrodes demonstrate that iodide has an oxidation peak at 720 mV, but the current is increasing starting from 660 mV (Figure 1(A)). The thiocholine oxidation peak is very wide and an overpotential as high as possible is necessary for a good sensitivity. Amperometric measurements made at 700 mV allowed a satisfactory sensitivity for thiocholine of 9.4 nA/μM with a limit of detection of 1 μM thiocholine. Nevertheless, at this potential it is not possible to use a substrate that contains iodide as counter anion because the parasite signals due to iodide are considerably larger. Thus, the signal obtained at the injection of 0.1 mM KI is 2,930 nA while for 25 mM thiocholine is measured only 218 nA. The analytical signal produced by the oxidation of iodide anions was reduced to 23 nA for 0.5 mM KI using a lower potential of 600 mV. At this potential, the thiocholine measurements sensitivity is significantly reduced in the absence of KI to only 3.59 nA/μM. Another interesting aspect is the fact that the thiocholine measurements sensitivity at 600 mV is further reduced in the presence 1 mM KI to 1.51 nA/μM. Our experiments carried out with screen-printed electrodes modified with multi-wall carbon nanotubes (MWCNT) demonstrates that the oxidation peaks of iodide (677 mV) is at a potential substantially higher in comparison with thiocholine (360 mV) (Figure 1(B)). This difference of oxidation potential allowed the detection of thiocholine at 360 mV without interferences from iodide anions. The sensitivity for thiocholine obtained using MWCNT screen-printed electrodes was 6.82 nA/μM in the absence of iodide. The sensitivity of MWCNT electrodes is lower than the carbon screen-printed electrodes obtained at 700 mV, but are avoided the interferences produced by iodide. All these data recommend the use of MWCNT electrodes for measurements with iodide containing substrate and carbon paste screen-printed electrodes for chloride containing substrate.

In the literature the difficulty of using the carbon based electrodes in combination with iodide containing enzymatic substrate was observed. Different strategies were used to address this problem like covering the WE surface with a Nafion layer that decreases the oxidation overpotential of the thiol moiety to 400 mV [11] or the use of WE made from epoxy-carbon composites covered with a cellulose nitrate enzymatic membrane to separate of the oxidation of thiocholine and iodide so that the current measured at 610 mV is mainly due only to the thiocholine [12]. Two reversible peaks were recorded by CV using WE made from spectrally pure graphite: one a lower potential one that was attributed to iodide and the second due to thiocholine at a potential that it is pH dependent [13]. The authors were able to obtain inhibition percentage by amperometric measurements at 800 mV (the higher potential), but were unable to observe a total inhibition of the enzyme because, besides the enzymatically produced thiocholine, they measured also the parasite signal of the iodide oxidation [14]. Other papers describing cholinesterase based biosensors that use iodide containing substrate without investigating the effect of iodide: home-made screen-printed electrodes using amperometric detection at +600 mV [15] or a glassy carbon modified with oxidized exfoliated graphite nanoplatelet (xGnPs) dispersed in chitosan with signal quantification by CV (peak at 950 mV) [16]. It is interesting to mention that iodide is not only an interfering substance that is simultaneously oxidized with thiocholine, but it acts also as a mediator for the oxidation of thiocholine [17]. This catalytic effect amplifies the difficulty of biosensor signal attribution and increases the measurement errors.

### 3.3. Gold Screen-Printed Electrodes

**Figure 2.**
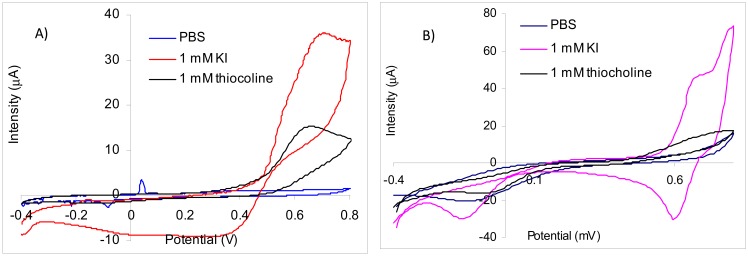
The cyclic voltammograms obtained in PBS, iodide and thiocholine solutions using: (**A**) gold screen-printed electrode. (**B**) platinum screen-printed electrode.

Using screen-printed gold electrodes it was obtained by CV for thiocholine a large irreversible peak with a maximum at 660 mV. The oxidation peak of iodide has the maximum at 695 mV, but the current increases starting from 390 mV (Figure 2(A)). Because the peaks for iodide and thiocholine appear at similar potentials and the iodide concentration is relatively high in comparison with thiocholine, it is not possible to measure the thiocholine in the presence of iodide at 660 mV. Thus, despite the fact that the sensitivity of the thiocholine measurements performed at 660 mV is 15.06 nA/μM in the absence of iodide, the signal recorded for the injection of 0.5 mM KI is 13,700 nA in comparison with only 702 nA obtained for 45 μM thiocholine. Nevertheless, we have observed a catalytic effect of iodide at lower potential: at 370 mV in the presence of 1 mM KI the sensitivity of the thiocholine measurements is 7.84 nA/μM. These results suggest that acetylthiocholine chloride may be easily used as substrate for AChE based biosensors constructed using screen-printed gold electrodes at 660 mV. The acetylthiocholine iodide may be used as substrate based on the catalytic properties of iodide at lower potentials, but only with a careful optimization of the working conditions to avoid false signals due to iodide oxidation.

The comparison with the experimental results reported in the literature is not straight forward because the gold electrodes or nanoparticles are usually modified with a self-assembled monolayer (SAM) made with different thiols or other organic layers for the enzyme immobilization. Thus, amperometric biosensors obtained by immobilization of acetylcholinesterase with EDC on gold disk electrodes modified with 3-mercaptopropionic acid were used at 280 mV, while the iodide peak (attributed by authors to “the oxidation of the substrate”) was 480 mV [18]. Using gold disposable electrochemical printed gold chips the enzyme free in the solution, two different peaks were recorded by differential pulse voltammetry (DPV) for tiocholine at 210 mV and respectively 270 mV while the iodide was oxidized around 500 mV [19]. The amperometric signal was recorded at 200 mV for gold electrodes modified with a SAM made from 11-mercaptoundecanoic acid (a long chain compound with passivating properties) [20].

The affinity of thiol moiety for the gold surfaces leading must be taken into consideration when one is using gold electrodes for the construction of cholinesterase based biosensors and acetylthiocholine as substrate. While the affinity thiocholine for gold provides an explanation for the improved sensitivity of the gold electrodes in comparison with carbon materials [19], the locally produced might be chemisorbed on the gold electrodes that are covered or not by a SAM. Based on this affinity, a colorimetric biosensor based on AChE inhibition was developed using the enzymatically produced thiocholine for the modulation of the growth of Au-Ag nanoparticles on seeding gold nanoparticles in the presence of ascorbic acid [21].

### 3.4. Platinum Screen-Printed Electrodes

The CV experiments carried out with platinum screen-printed electrodes demonstrate that thiocholine oxidation is irreversible with a very large peak while the iodide cvasireversible oxidation peak had a maximum at 740 mV (Figure 2(B)). The current recorded for the oxidation of thiocholine increases from 300 mV, but for a better sensitivity is necessary to use a higher potential. The sensitivity for thiocholine amperometric measurements at 700 mV was 27.9 nA/μM, but the signals produced by iodide at this potential are very high. The iodide oxidation starts from 570 mV. The inhibition measurements are performed in a three steps procedure that separates the amperometric quantification of the enzyme activity using standard solution form the biosensor incubation in the real sample [1] and, in consequence, the electrochemical interferences from sample matrix are avoided. Thus, we have tested a potential of 560 mV for the amperometric measurements in the presence of iodide using platinum electrodes. At this potential, the sensitivity for thiocholine amperometric detection was 18.56 nA/μM and only minimum interferences from iodide anion was observed. These results demonstrate the possibility to use acetylthiocholine iodide as substrate for AChE based biosensors only at low potentials to avoid the iodide oxidation.

Acetylthiocholine iodide was used as substrate for biosensors based on AChE immobilized on platinum electrodes poised at 410 mV (a lower potential than the maximum theoretical) [22]. A potentiometric method for the quantification of AChE activity is based on the modification of the potential recorded for the [I_2_]/[I^−^]^2^ couple by the more easily oxidizable thiocholine. It is interesting to note that this potentiometric method needs an electroactive compound in the solution [23] and, in consequence, substrate containing chloride anion is not usable.

### 3.5. Cobalt Phthalocyanine Screen-Printed Electrodes

**Figure 3.**
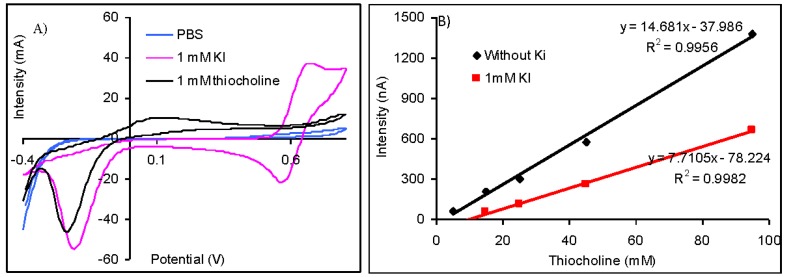
The results obtained using cobalt phthalocyanine screen-printed electrodes using: (**A**) cyclic voltammograms obtained in PBS, iodide and thiocholine solutions. (**B**) amperometric signals obtained for the measurement of thiocholine in the absence and presence of 0.6 mM KI.

The difference between the potential of the oxidation peak of thiocholine at 110 mV and iodide at 675 mV is important (Figure 3(A)) and suggest the possibility to quantify the thiolic compounds without interferences from iodide anions. Nevertheless, the amperometric measurements made at 110 mV demonstrated that the sensitivity for the thiocholine detection is reduced from 14.68 nA/μM (obtained without iodide) to only 7.71 nA/μM (in the case of measurements carried out in solutions containing 1 mM KI). The injection of 1 mM KI does not produce a significant oxidation current, but it reduces the magnitude of the analytical signals (Figure 3(B)). Despite the fact that it is possible to quantify the analytical signals of AChE based biosensors using acetylthiocholine iodide as substrate, it is recommendable to use acetylthiocholine chloride due to the lower sensibility that is achieved in the presence of iodide anions.

Different potentials were reported in literature for the quantification of the analytical signals of biosensors developed by the immobilization of AChE on electrodes modified with cobalt phthalocyanine when acetylthiocholine iodide was used as substrate. A wide oxidation peak attributed by the authors to the overlap of the peaks of unknown species was obtained by differential pulse voltammetry recorded from 100 to 1,000 mV. An oxidation peak attributed to the thiocholine with maximum at 370 mV with a height approximately equal with one third of the previous wide peak was obtained when the differential pulse voltammetry was carried out in a narrower potential range between 100 and 500 mV. The optimum potential for amperometric measurements chosen by authors was 370 mV, but the enzymatic inhibition may be determined between 300 and 400 mV [24]. The thiocholine was measured without interferences from iodide at a lower potential of only 100 mV that was chosen by hydrodynamic cyclic voltammetry, but authors caution on the important differences between their results and the ones obtained by other teams that may be due to variation in electrode material and possible electrode conditioning [25].

### 3.6. Tests Carried Out with Biosensors

The experiments described above are useful to predict the behavior of biosensors constructed by the immobilization of AChE on the surface of similar electrodes. Nevertheless, the electrode surface is modified in the case biosensors and in consequence the electrochemical propertied may be modified. We have verified our results using biosensors obtain by the immobilization of AChE by reticulation with glutaraldehyde on the surface of platinum and cobalt phthalocyanine screen-printed electrodes and the analytical signals were recorded for the injection of 1 mM acetylthiocholine chloride and iodide, respectively. The biosensors based on platinum screen-printed electrodes were tested at 560 mV and 700 mV. The response measured at 560 mV was 132 nA for both chloride and iodide substrates. The response obtained at 700 mV was 531 nA using acetylthiocholine iodide much higher in comparison with 148 nA that were recorded for acetylthiocholine chloride. The immobilized enzyme was completely denatured by incubation for 3 min in a 1:1 water-methanol solution and the residual response was recorded in order to test the interferences. At 560 mV it was not measured any significant signal for the injection of both chloride and iodide, but at 700 mV the response measured for acetylthiocholine iodide is 389 nA while for acetylthiocholine iodide the signal is still insignificant. For the biosensors based on cobalt phthalocyanine screen-printed electrodes it was obtained at 110 mV an analytical signal of 156 nA for acetylthiocholine chloride that is statistically higher than 118 nA recorded for acetylthiocholine chloride. All these results confirm the conclusions reached using bare electrodes, but tests with chloride and iodide substrate must be carried out for every particular developed biosensor in order to avoid false signals.

## 4. Conclusions

Our study demonstrates that it is possible to use acetylthiocholine iodide as pseudosubstrate for the quantification of the analytical signals of amperometric biosensors based on AChE only after a careful investigation of the electrochemical behavior of the electrode towards thiocholine in the presence of iodide. Iodide anions may interfere with the measurements in two different ways: (i) by the modification of the measurement sensibility of the thiocholine and/or (ii) by producing false analytical signal due to the oxidation. The influence of the iodide on the measurements and optimum potential must be determined experimentally for every developed biosensor because each layer deposed on the electrode surface modifies the overall electrochemical behavior.
